# 4-(4-Chloro­phen­yl)piperidin-4-ol

**DOI:** 10.1107/S1600536810004216

**Published:** 2010-02-06

**Authors:** Grzegorz Dutkiewicz, B. P. Siddaraju, H. S. Yathirajan, M. S. Siddegowda, Maciej Kubicki

**Affiliations:** a Department of Chemistry, Adam Mickiewicz University, Grunwaldzka 6, 60-780 Poznań, Poland; b Department of Chemistry, V. V. Puram College of Science, Bangalore 560 004, India; c Department of Studies in Chemistry, University of Mysore, Manasagangotri, Mysore 570 006, India

## Abstract

In the title compound, C_11_H_14_ClNO, the piperidine ring adopts a chair conformation: the hydroxyl substituent and the N-bound H atom occupy the axial positions, while the benzene ring occupies the equatorial position. In the crystal, the mol­ecules are linked into a centrosymmetric tetra­mer through strong O—H⋯N and weak N—H⋯O hydrogen bonds; the N and O atoms act as both donor and acceptor for these inter­actions. The tetra­mers are further joined by hydrogen bonds into a layer parallel to (100).

## Related literature

For related structures, see: De Camp & Ahmed (1972*a* ▶,*b* ▶); Friederich *et al.* (1993 ▶); Kimura & Okabayashi (1986 ▶). For details of the asymmetry parameters for chair conformations, see: Duax & Norton (1975 ▶). For a description of the Cambridge Structural Database, see: Allen (2002 ▶).
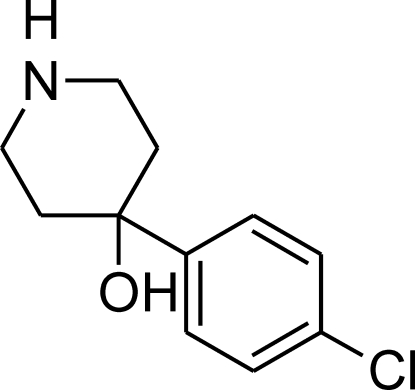


## Experimental

### 

#### Crystal data

C_11_H_14_ClNO
*M* *_r_* = 211.68
Monoclinic, 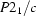

*a* = 11.3706 (10) Å
*b* = 9.5204 (8) Å
*c* = 10.6164 (9) Å
β = 108.458 (8)°
*V* = 1090.13 (16) Å^3^
*Z* = 4
Cu *K*α radiation
μ = 2.83 mm^−1^
*T* = 295 K
0.3 × 0.2 × 0.15 mm

#### Data collection

Oxford Diffraction SuperNova, single source at offset, Atlas diffractometer
Absorption correction: multi-scan (*CrysAlis PRO*; Oxford Diffraction, 2009 ▶) *T*_min_ = 0.401, *T*_max_ = 0.654
4068 measured reflections
2190 independent reflections
2014 reflections with *I* > 2σ(*I*)
*R*_int_ = 0.011

#### Refinement

*R*[*F* ^2^ > 2σ(*F* ^2^)] = 0.038
*wR*(*F* ^2^) = 0.111
*S* = 1.07
2190 reflections
183 parameters
H atoms treated by a mixture of independent and constrained refinement
Δρ_max_ = 0.32 e Å^−3^
Δρ_min_ = −0.38 e Å^−3^

### 

Data collection: *CrysAlis PRO* (Oxford Diffraction, 2009 ▶); cell refinement: *CrysAlis PRO*; data reduction: *CrysAlis PRO*; program(s) used to solve structure: *SIR92* (Altomare *et al.*, 1993 ▶); program(s) used to refine structure: *SHELXL97* (Sheldrick, 2008 ▶); molecular graphics: *Stereochemical Workstation Operation Manual* (Siemens, 1989 ▶) and *Mercury* (Macrae *et al.*, 2008 ▶); software used to prepare material for publication: *SHELXL97*.

## Supplementary Material

Crystal structure: contains datablocks I, global. DOI: 10.1107/S1600536810004216/is2520sup1.cif

Structure factors: contains datablocks I. DOI: 10.1107/S1600536810004216/is2520Isup2.hkl

Additional supplementary materials:  crystallographic information; 3D view; checkCIF report

## Figures and Tables

**Table 1:** Hydrogen-bond geometry (Å, °)

*D*—H⋯*A*	*D*—H	H⋯*A*	*D*⋯*A*	*D*—H⋯*A*
N1—H1⋯O4^i^	0.89 (2)	2.41 (2)	3.2036 (16)	147.2 (17)
O4—H4*A*⋯N1^ii^	0.84 (2)	1.97 (2)	2.8089 (17)	174 (2)

Symmetry codes: (i) 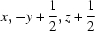; (ii) 
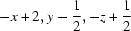
.

**Fig. 1.**
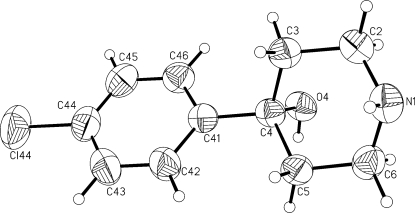
Anisotropic ellipsoid representation of the compound 1 together with atom labelling scheme. The ellipsoids are drawn at 50% probability level, hydrogen atoms are depicted as spheres with arbitrary radii.

**Fig. 2.**
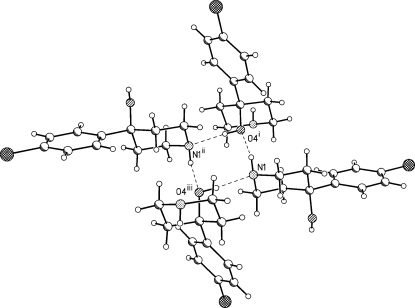
Hydrogen-bonded tetramer [symmetry codes: (i) x, 1/2 - y, 1/2 + z; (ii) 2 - x, 1 - y, 1 - z; (iii) 2 - x, 1/2 + y, 1/2 - z].

**Fig. 3.**
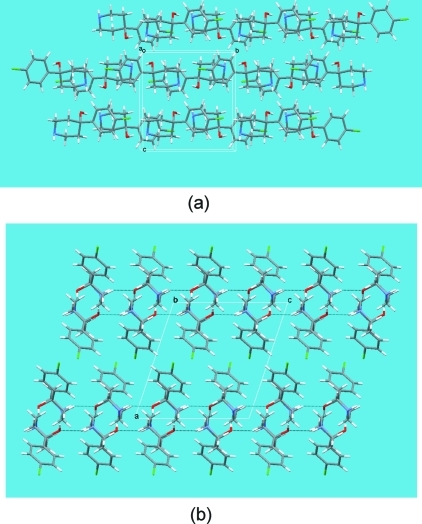
The packing of the molecules of 1. (a) Hydrogen-boded layer; (b) the packing as seen along the y-direction.

## supplementary crystallographic information

### Comment

The title compound, (1, Scheme 1), 4-(4-chlorophenyl)piperidin-4-ol is used as an intermediate for the synthesis of pharmaceuticals such as haloperidol (neuroleptic drug used to treat psychotic illnesses, extreme agitation, or Tourette's syndrome) and loperamide which is effective against diarrhea resulting from gastroenteritis or inflammatory bowel disease.

The piperidine ring adopts an almost ideal chair conformation (Fig. 1); the asymmetry parameters (Duax & Norton, 1975) are all smaller than 2.5°. The hydroxy group and N—H hydrogen atom occupy the axial positions [torsion angles: C2—C3—C4—O4 -64.46 (15)°, C6—C5—C4—O4 60.81 (15)°, C5—C6—N1—H1 65.0 (13)°, and C3—C2—N1—H1 -64.8 (14)°]. Such a mutual conformation of hydroxyl and phenyl groups is very typical, in the Cambridge Database (Allen, 2002; ver. 5.30 of Nov. 2008, last update Sep. 2009) there are 65 crystal structures of six-membered saturated rings with both OH and aromatic substituent in one position, only in three of them the hydroxyl group adopts the equatorial position [two polymorphs of (±)-β-1,2,5-trimethyl-4-phenylpiperidin-4-ol (De Camp & Ahmed, 1972*a*,*b*), cis-1,4-bis(4-bromophenyl)-1,4-dimethoxycyclohexane (Friederich *et al.*, 1993), and cis-1-phenyl-3-piperidinocyclohexan-1-ol hydrochloride (Kimura & Okabayashi, 1986)].

The relatively strong and directional O—H···N hydrogen bonds join the molecules of 1, related by two-fold screw axis, into the chains along [010] directions. These chains are interconnected by far weaker N—H···O hydrogen bonds. These two kinds of contacts form centrosymmetric tetramers of the molecules (Fig. 2). In the crystal structures there are the hydrogen-bonded layers of molecules, created by interconnecting chains, in the *bc* plane (Fig. 3a). There are no directional interactions between neighbouring layers (Fig. 3b).

### Experimental

The title compound was obtained as a gift sample from R. L. Fine Chem, Bangalore, India. X-ray quality crystals were obtained by a slow evaporation from an ethyl acetate solution (m.p. 410–413 K).

### Refinement

Hydrogen atoms were found in the subsequent difference Fourier maps, and freely refined.

### Crystal data

**Table:**

C_11_H_14_ClNO	*F*(000) = 448
*M**_r_* = 211.68	*D*_x_ = 1.290 Mg m^−^^3^
Monoclinic, *P*2_1_/*c*	Cu *K*α radiation, λ = 1.54178 Å
Hall symbol: -P 2ybc	Cell parameters from 3304 reflections
*a* = 11.3706 (10) Å	θ = 4.1–75.2°
*b* = 9.5204 (8) Å	µ = 2.83 mm^−^^1^
*c* = 10.6164 (9) Å	*T* = 295 K
β = 108.458 (8)°	Prism, yellow
*V* = 1090.13 (16) Å^3^	0.3 × 0.2 × 0.15 mm
*Z* = 4	

### Data collection

**Table:**

Oxford Diffraction SuperNova, single source at offset, Atlas diffractometer	2190 independent reflections
Radiation source: SuperNova (Cu) X-ray Source	2014 reflections with *I* > 2σ(*I*)
mirror	*R*_int_ = 0.011
Detector resolution: 10.5357 pixels mm^-1^	θ_max_ = 75.3°, θ_min_ = 4.1°
ω scans	*h* = −13→14
Absorption correction: multi-scan (*CrysAlis PRO*; Oxford Diffraction, 2009)	*k* = −11→7
*T*_min_ = 0.401, *T*_max_ = 0.654	*l* = −12→13
4068 measured reflections	

### Refinement

**Table:**

Refinement on *F*^2^	Primary atom site location: structure-invariant direct methods
Least-squares matrix: full	Secondary atom site location: difference Fourier map
*R*[*F*^2^ > 2σ(*F*^2^)] = 0.038	Hydrogen site location: inferred from neighbouring sites
*wR*(*F*^2^) = 0.111	H atoms treated by a mixture of independent and constrained refinement
*S* = 1.07	*w* = 1/[σ^2^(*F*_o_^2^) + (0.0549*P*)^2^ + 0.250*P*] where *P* = (*F*_o_^2^ + 2*F*_c_^2^)/3
2190 reflections	(Δ/σ)_max_ < 0.001
183 parameters	Δρ_max_ = 0.32 e Å^−^^3^
0 restraints	Δρ_min_ = −0.38 e Å^−^^3^

### Special details

**Table:**

Geometry. All s.u.'s (except the s.u. in the dihedral angle between two l.s. planes) are estimated using the full covariance matrix. The cell s.u.'s are taken into account individually in the estimation of s.u.'s in distances, angles and torsion angles; correlations between s.u.'s in cell parameters are only used when they are defined by crystal symmetry. An approximate (isotropic) treatment of cell s.u.'s is used for estimating s.u.'s involving l.s. planes.
Refinement. Refinement of *F*^2^ against ALL reflections. The weighted *R*-factor wR and goodness of fit *S* are based on *F*^2^, conventional *R*-factors *R* are based on *F*, with *F* set to zero for negative *F*^2^. The threshold expression of *F*^2^ > 2σ(*F*^2^) is used only for calculating *R*-factors(gt) etc. and is not relevant to the choice of reflections for refinement. *R*-factors based on *F*^2^ are statistically about twice as large as those based on *F*, and *R*- factors based on ALL data will be even larger.

### Fractional atomic coordinates and isotropic or equivalent isotropic displacement parameters (Å^2^)

**Table:**

	*x*	*y*	*z*	*U*_iso_*/*U*_eq_	
N1	0.92832 (13)	0.42199 (14)	0.33836 (13)	0.0530 (3)	
H1	0.8930 (19)	0.430 (2)	0.402 (2)	0.069 (5)*	
C2	0.83116 (16)	0.40784 (17)	0.20917 (16)	0.0537 (4)	
H21	0.8738 (17)	0.4069 (19)	0.1413 (18)	0.058 (5)*	
H22	0.7767 (18)	0.491 (2)	0.1973 (18)	0.065 (5)*	
C3	0.75467 (14)	0.27404 (16)	0.19461 (16)	0.0507 (3)	
H31	0.7086 (19)	0.277 (2)	0.255 (2)	0.072 (6)*	
H32	0.6977 (17)	0.2675 (19)	0.0997 (19)	0.061 (5)*	
C4	0.83678 (12)	0.14272 (14)	0.22350 (12)	0.0414 (3)	
O4	0.89540 (10)	0.13797 (11)	0.12224 (9)	0.0476 (3)	
H4A	0.949 (2)	0.073 (2)	0.140 (2)	0.074 (6)*	
C5	0.93538 (14)	0.16101 (17)	0.36010 (13)	0.0462 (3)	
H51	0.9930 (16)	0.0777 (19)	0.3783 (16)	0.052 (4)*	
H52	0.8963 (17)	0.1649 (19)	0.4293 (18)	0.058 (5)*	
C6	1.00732 (15)	0.29679 (18)	0.36784 (15)	0.0534 (4)	
H61	1.0557 (17)	0.2932 (19)	0.3036 (18)	0.059 (5)*	
H62	1.0676 (18)	0.307 (2)	0.457 (2)	0.066 (5)*	
C41	0.75873 (12)	0.01087 (15)	0.21490 (13)	0.0433 (3)	
C42	0.76509 (17)	−0.0736 (2)	0.32296 (16)	0.0604 (4)	
H42	0.818 (2)	−0.051 (2)	0.409 (2)	0.080 (6)*	
C43	0.69364 (19)	−0.1934 (2)	0.3105 (2)	0.0715 (5)	
H43	0.699 (2)	−0.251 (2)	0.384 (2)	0.084 (6)*	
C44	0.61247 (15)	−0.22890 (18)	0.18927 (19)	0.0600 (4)	
Cl44	0.52022 (5)	−0.37870 (6)	0.17414 (7)	0.0901 (2)	
C45	0.60313 (16)	−0.14761 (19)	0.07924 (18)	0.0601 (4)	
H45	0.550 (2)	−0.174 (2)	−0.007 (2)	0.081 (6)*	
C46	0.67646 (14)	−0.02996 (18)	0.09244 (15)	0.0536 (4)	
H46	0.6735 (18)	0.028 (2)	0.014 (2)	0.072 (6)*	

### Atomic displacement parameters (Å^2^)

**Table:**

	*U*^11^	*U*^22^	*U*^33^	*U*^12^	*U*^13^	*U*^23^
N1	0.0619 (8)	0.0540 (7)	0.0485 (7)	−0.0073 (6)	0.0250 (6)	−0.0050 (5)
C2	0.0587 (9)	0.0478 (8)	0.0548 (8)	0.0021 (7)	0.0181 (7)	0.0037 (6)
C3	0.0468 (7)	0.0509 (8)	0.0539 (8)	0.0038 (6)	0.0152 (6)	0.0030 (6)
C4	0.0446 (7)	0.0485 (7)	0.0339 (6)	0.0023 (5)	0.0163 (5)	0.0033 (5)
O4	0.0558 (6)	0.0550 (6)	0.0377 (5)	0.0044 (5)	0.0227 (4)	0.0064 (4)
C5	0.0492 (7)	0.0539 (8)	0.0356 (6)	−0.0010 (6)	0.0136 (5)	0.0032 (5)
C6	0.0508 (8)	0.0627 (9)	0.0451 (7)	−0.0073 (7)	0.0129 (6)	−0.0003 (7)
C41	0.0435 (6)	0.0479 (7)	0.0407 (6)	0.0032 (6)	0.0167 (5)	0.0004 (5)
C42	0.0649 (10)	0.0668 (10)	0.0459 (8)	−0.0133 (8)	0.0123 (7)	0.0086 (7)
C43	0.0746 (11)	0.0722 (12)	0.0672 (10)	−0.0159 (9)	0.0216 (9)	0.0162 (9)
C44	0.0495 (8)	0.0533 (9)	0.0805 (11)	−0.0037 (7)	0.0253 (8)	−0.0041 (8)
Cl44	0.0732 (3)	0.0695 (3)	0.1295 (5)	−0.0240 (2)	0.0349 (3)	−0.0071 (3)
C45	0.0515 (8)	0.0656 (10)	0.0609 (9)	−0.0039 (7)	0.0145 (7)	−0.0124 (8)
C46	0.0540 (8)	0.0600 (9)	0.0453 (7)	−0.0006 (7)	0.0138 (6)	−0.0004 (7)

### Geometric parameters (Å, °)

**Table:**

N1—C6	1.465 (2)	C5—H52	0.972 (19)
N1—C2	1.470 (2)	C6—H61	1.003 (19)
N1—H1	0.89 (2)	C6—H62	0.99 (2)
C2—C3	1.522 (2)	C41—C42	1.384 (2)
C2—H21	0.987 (19)	C41—C46	1.395 (2)
C2—H22	0.99 (2)	C42—C43	1.382 (3)
C3—C4	1.5322 (19)	C42—H42	0.95 (2)
C3—H31	0.95 (2)	C43—C44	1.368 (3)
C3—H32	1.013 (18)	C43—H43	0.94 (2)
C4—O4	1.4337 (15)	C44—C45	1.377 (3)
C4—C41	1.5233 (19)	C44—Cl44	1.7473 (17)
C4—C5	1.5365 (18)	C45—C46	1.377 (2)
O4—H4A	0.84 (2)	C45—H45	0.96 (2)
C5—C6	1.518 (2)	C46—H46	0.99 (2)
C5—H51	1.008 (17)		
			
C6—N1—C2	110.71 (12)	C4—C5—H52	110.2 (11)
C6—N1—H1	107.4 (13)	H51—C5—H52	108.1 (14)
C2—N1—H1	109.3 (13)	N1—C6—C5	113.44 (13)
N1—C2—C3	114.07 (13)	N1—C6—H61	108.3 (10)
N1—C2—H21	106.5 (10)	C5—C6—H61	109.6 (10)
C3—C2—H21	108.4 (11)	N1—C6—H62	108.6 (11)
N1—C2—H22	107.6 (11)	C5—C6—H62	109.5 (11)
C3—C2—H22	110.0 (11)	H61—C6—H62	107.2 (15)
H21—C2—H22	110.3 (15)	C42—C41—C46	117.02 (14)
C2—C3—C4	111.72 (12)	C42—C41—C4	123.54 (13)
C2—C3—H31	109.2 (13)	C46—C41—C4	119.44 (12)
C4—C3—H31	108.6 (12)	C43—C42—C41	121.70 (15)
C2—C3—H32	108.5 (10)	C43—C42—H42	117.1 (13)
C4—C3—H32	107.8 (10)	C41—C42—H42	121.2 (13)
H31—C3—H32	111.1 (15)	C44—C43—C42	119.67 (16)
O4—C4—C41	109.23 (10)	C44—C43—H43	119.2 (14)
O4—C4—C3	105.91 (11)	C42—C43—H43	121.1 (14)
C41—C4—C3	110.72 (11)	C43—C44—C45	120.47 (16)
O4—C4—C5	109.74 (11)	C43—C44—Cl44	119.66 (14)
C41—C4—C5	112.78 (11)	C45—C44—Cl44	119.87 (14)
C3—C4—C5	108.23 (12)	C46—C45—C44	119.30 (15)
C4—O4—H4A	109.3 (14)	C46—C45—H45	119.4 (13)
C6—C5—C4	111.55 (12)	C44—C45—H45	121.3 (13)
C6—C5—H51	110.7 (10)	C45—C46—C41	121.82 (15)
C4—C5—H51	109.1 (9)	C45—C46—H46	120.8 (12)
C6—C5—H52	107.1 (11)	C41—C46—H46	117.4 (12)
			
C6—N1—C2—C3	53.23 (17)	O4—C4—C41—C46	−49.35 (16)
N1—C2—C3—C4	−54.44 (18)	C3—C4—C41—C46	66.91 (16)
C2—C3—C4—O4	−64.46 (15)	C5—C4—C41—C46	−171.65 (13)
C2—C3—C4—C41	177.25 (12)	C46—C41—C42—C43	0.2 (3)
C2—C3—C4—C5	53.16 (15)	C4—C41—C42—C43	−179.21 (17)
O4—C4—C5—C6	60.81 (15)	C41—C42—C43—C44	−1.3 (3)
C41—C4—C5—C6	−177.17 (11)	C42—C43—C44—C45	1.2 (3)
C3—C4—C5—C6	−54.33 (15)	C42—C43—C44—Cl44	−178.91 (16)
C2—N1—C6—C5	−54.19 (16)	C43—C44—C45—C46	0.0 (3)
C4—C5—C6—N1	56.56 (16)	Cl44—C44—C45—C46	−179.90 (13)
O4—C4—C41—C42	130.07 (15)	C44—C45—C46—C41	−1.1 (3)
C3—C4—C41—C42	−113.68 (16)	C42—C41—C46—C45	1.0 (2)
C5—C4—C41—C42	7.76 (19)	C4—C41—C46—C45	−179.54 (14)

### Hydrogen-bond geometry (Å, °)

**Table:**

*D*—H···*A*	*D*—H	H···*A*	*D*···*A*	*D*—H···*A*
N1—H1···O4^i^	0.89 (2)	2.41 (2)	3.2036 (16)	147.2 (17)
O4—H4A···N1^ii^	0.84 (2)	1.97 (2)	2.8089 (17)	174 (2)

Symmetry codes: (i) *x*, −*y*+1/2, *z*+1/2; (ii) −*x*+2, *y*−1/2, −*z*+1/2.
